# Prognostication of Half-Life Clearance of Plasma EBV DNA in Previously Untreated Non-metastatic Nasopharyngeal Carcinoma Treated With Radical Intensity-Modulated Radiation Therapy

**DOI:** 10.3389/fonc.2020.01417

**Published:** 2020-08-21

**Authors:** Sik-Kwan Chan, Sum-Yin Chan, Horace Cheuk-Wai Choi, Chi-Chung Tong, Ka-On Lam, Dora Lai-Wan Kwong, Varut Vardhanabhuti, To-Wai Leung, Mai-Yee Luk, Anne Wing-Mui Lee, Victor Ho-Fun Lee

**Affiliations:** ^1^Department of Clinical Oncology, Li Ka Shing Faculty of Medicine, The University of Hong Kong, Hong Kong, China; ^2^Clinical Oncology Center, The University of Hong Kong-Shenzhen Hospital, Shenzhen, China; ^3^Department of Diagnostic Radiology, Li Ka Shing Faculty of Medicine, The University of Hong Kong, Hong Kong, China

**Keywords:** nasopharyngeal carcinoma, intensity-modulated radiation therapy, plasma Epstein–Barr virus deoxyribonucleic acid, half-life clearance, prognostication

## Abstract

**Introduction:** The prognostic role of plasma Epstein–Barr virus (EBV) DNA clearance when intensity-modulated radiotherapy (IMRT) and the 8th edition of American Joint Committee on Cancer (AJCC)/Union for International Cancer Control (UICC) TNM Staging Classification are fully implemented remains undeciphered. We investigated if its half-life clearance during radical treatment for non-metastatic nasopharyngeal carcinoma (NPC) was an early prognosticator.

**Patients and methods:** Patients with previously untreated non-metastatic NPC were prospectively treated with radical IMRT and concurrent chemotherapy +/– induction/adjuvant chemotherapy from 2014 to 2018. Their plasma EBV DNA was measured immediately before treatment followed by weekly schedules until 0 copy/ml in two consecutive measurements. Cox regression models were employed to identify prognostic factors.

**Results:** Forty-five patients were prospectively recruited and analyzed. After a median follow-up of 30.3 months, 2 (4.5%), 1 (2.3%), and 6 (13.6%) patients experienced local, regional, and distant relapses, respectively. The median half-life clearance of plasma EBV DNA was 7.92 days. Those with half-life clearance of >15 days had a worse 3-years progression-free survival (PFS) (79.5 vs. 25.0%, *p* = 0.005), distant metastasis-free survival (DMFS) (85.0 vs. 31.3%, *p* = 0.009), and overall survival (OS) (91.3 vs. 75.0%, *p* = 0.024) when compared to those with a shorter half-life. Multivariable analyses demonstrated that only half-life (>15 days) was prognostic of DMFS [HR (95% CI): 4.91 (1.31; 18.39), *p* = 0.01] and OS [HR (95% CI): 5.24 (1.06; 26.05)] while half-life (>15 days) [HR (95% CI): 5.14 (1.28; 22.73), *p* = 0.02] and sum of pretreatment gross tumor volumes of the primary nasopharyngeal tumor and the radiologically positive neck nodes (GTV_P+N) [HR (95% CI): 1.01 (1.00; 1.03), *p* = 0.02] were prognostic of PFS.

**Conclusion:** The half-life clearance of plasma EBV DNA was prognostic in non-metastatic NPC staged and treated in the contemporary era. Earlier biomarker surveillance during treatment should be considered.

**Clinical Trial Registration:** This study has been registered with ClinicalTrials.gov (Identifier: NCT03830996).

## Introduction

Nasopharyngeal carcinoma (NPC) is endemic in Southeast Asia including Hong Kong (1). Radiation therapy alone is the standard of care for early-stage disease, while concurrent chemoradiation is indicated for locoregionally advanced stage III–IVB disease. Intensity-modulated radiation therapy (IMRT) has been the most effective and widely adopted contemporary technique. In virtue of its superior target coverage and dose sparing to adjacent critical organs at risks, IMRT produces better treatment outcomes and toxicity profiles when compared to the traditional techniques (1–3). Indeed, the latest 8th edition of the American Joint Committee on Cancer (AJCC)/Union for International Cancer Control (UICC) Staging Classification (TNM) relies on the improved locoregional control by IMRT, apart from more detailed pretreatment imaging with magnetic resonance imaging (MRI) leading to more homogeneous definitions of T2 (vs. T4) and N3 disease (4).

Plasma Epstein–Barr Virus (EBV) deoxyribonucleic acid (DNA) at baseline, during treatment, and after treatment has long been investigated and advocated as a surrogate marker for detection, monitoring, and prognostication of NPC (5–11). In addition, kinetics of plasma EBV DNA during radiation therapy was studied, and its clearance rate was demonstrated as a prognostic factor for previously untreated or recurrent NPC (12–15). Its clearance and prognostic role after salvage surgery for recurrent NPC was also investigated (16, 17). All of these studies were conducted long time ago when old radiation techniques and the earlier editions of AJCC/UICC staging classification were still used. Although the prognostic roles of plasma EBV DNA measured at the beginning, in the midcourse, and after treatment have been evaluated in IMRT era, there has been hitherto no publications with reference to the impact of its half-time clearance (which necessitates more frequent measurement) on survival in patients staged and treated in IMRT era and the current edition of AJCC/UICC staging classification (18–23).

There is definitely an unmet and urgent need for exploring the possibility of shifting the prognosticative role of plasma EBV DNA from posttreatment titers to earlier and more frequent measurement to detect its clearance during the initial course of radical treatment. This will certainly enable us to identify and detect early poor responders leading to more prompt investigations and better subsequent personalized and intensified treatment to reduce the chance of early relapse. We therefore initiated this prospective observational study on measuring baseline and weekly plasma EBV DNA titers since the inception of radical treatment for all patients with newly diagnosed non-metastatic NPC, to investigate the prognostic value of half-life of plasma EBV DNA clearance on survival outcomes. This study was registered with ClinicalTrials.gov (identifier NCT03830996).

## Patients and Methods

### Patient Population and Treatment

Patients with newly diagnosed histologically confirmed non-metastatic NPC under the care of the Department of Clinical Oncology at the Queen Mary Hospital in Hong Kong were recruited into this prospective observational study between May 2014 and July 2018. This study was approved by the local institutional review board (Institutional Review Board of the University of Hong Kong/Hospital Authority Hong Kong West Cluster, reference number UW 16-428 and UW 19-157) and undertaken according to the guidelines of Declaration of Helsinki and Good Clinical Practice. All patients, after detailed explanation by the study investigators, provided written informed consent before study commencement. They then had comprehensive pretreatment investigations including positron-emission tomography with integrated contrast-enhanced computed tomography (PET-CT) scan, magnetic resonance imaging (MRI) of the head and neck region, serum hematology, and biochemistry, as well as plasma EBV DNA as complete staging workup. Those with radiologically and/or histologically confirmed distant metastasis were excluded from this study. Staging was performed blindly and independently by one oncologist and one radiologist based on the 7th edition of AJCC/UICC TNM Staging Classification for treatment decision. Restaging was performed again based on the 8th edition of AJCC/UICC TNM Staging Classification for subsequent analyses in this study. Any stage discrepancy was resolved by consensus. IMRT alone was given for stage I or small-volume stage II disease, while cisplatin-based concurrent chemoradiation with or without adjunct chemotherapy (induction or adjuvant) was given for stage II disease with bulky ipsilateral nodal metastasis (lymph node size ≥3 cm) as well as stage III–IVA disease. The treatment details and follow-up surveillance as routine standard practice of all patients in our institution were described previously (Supplementary Data) (19).

Since treatment commencement, all patients had their EBV DNA titers measured at weekly intervals until it was undetectable in two consecutive measurements. The details of the extraction and enumeration of plasma EBV DNA were the one devised by Lo et al., which was also described in our previous publications (Supplementary Data) (5, 19, 24). In brief, all patient blood samples contained in ethylenediaminetetraacetic acid (EDTA) tubes were immediately stored in a 4°C refrigerator after blood taking from patients, and they were processed for subsequent EBV DNA extraction within 4 h of blood taking in the single laboratory of our institution (further details on EBV DNA quantification and validation methods were described in the Supplementary Data). A total of about 400–800 μl of plasma samples were used for DNA extraction by a QIAamp Blood Kit (Qiagen, Hilden, Germany). The exact amount of plasma was determined for the calculation of EBV DNA genome copies. Circulating EBV DNA concentrations were measured using a real-time quantitative polymerase chain reaction (PCR) system with ABI Prism® 7000 Sequence Detection System (Applied Biosystems, USA) that amplified a DNA segment in the *Bam*HI-W fragment region of the EBV genome. All samples were repeated twice on the same day by the same assay for accurate quantification, and the results showed that the discrepancy was <2% for all repeated samples. The results were expressed as EBV DNA genome copies per milliliter with accuracy to the nearest 0.1 copies/ml (24). Undetectable plasma EBV DNA meant 0 copies/ml, and they were used interchangeably in the main text and the Supplementary Material. Our study compiled with the REMARK recommendations for tumor marker prognostic studies using biological material (Supplementary Data).

## Statistical Analysis

Ratios of weekly plasma EBV DNA titers to their baseline were transformed into the corresponding natural logarithm values. In view of the use of logarithmic transformation, undetectable level of plasma EBV DNA was recoded from 0 to 0.1. Assuming an exponential model, a slope of –k was obtained when the natural logarithms were plotted against time by linear regression (25). The half-life was then determined by the equation of half-life = In2/k.

We investigated the extent to which the half-life of plasma EBV DNA clearance impinged on survival via its presumed association with progression of disease. To select the cutoff value for half-life of plasma EBV DNA clearance, receiver operating characteristic (ROC) curve was generated. Area under the ROC curve (AUC) and trade-off between sensitivity and specificity determined the optimal cut-off value of the half-life clearance of plasma EBV DNA to predict disease progression. Discrete categorical variables were compared by chi square tests or Fisher's exact tests whenever appropriate, while continuous variables were compared by Mann–Whitney *U*-tests.

The prespecified survival end points in this study included distant metastasis free-survival (DMFS), progression-free survival (PFS), and overall survival (OS). Kaplan–Meier estimation of survival outcomes and log-rank tests were employed for unadjusted comparisons of survival differences between patients with different half-life of plasma EBV DNA clearance. Univariable and multivariable analyses were performed by Cox proportional hazard models to identify the prognostic factors for these survival end points with age, gender, T-classification, N-classification, overall stage of NPC, pretreatment gross tumor volumes (GTVs) of the primary nasopharyngeal tumor (GTV_P), and the radiologically positive neck nodes (GTV_N), sum of GTV_P and GTV_N (GTV_P+N), and specific half-life of plasma EBV DNA clearance as covariates.

All statistical analyses were performed by Statistical Package for the Social Sciences (SPSS) version 25.0. All tests were two-sided, and *p* < 0.05 were considered statistically significant. The database-lock date for analysis was June 15, 2020.

## Results

**Figure 1:**
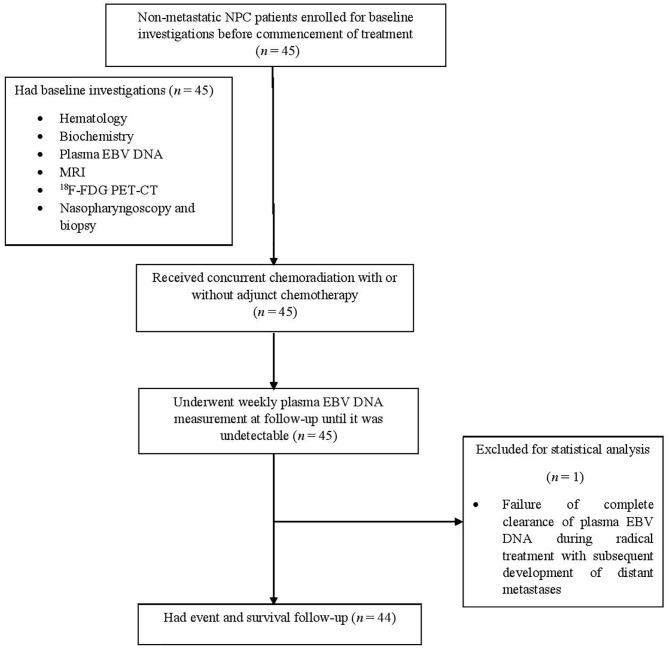
Study flowchart.

The study flowchart is shown in Figure 1. From May 2014 to September 2018, 45 patients were prospectively recruited in this study with their dispositions shown (Table 1). One (2.2%) patient was excluded in subsequent statistical analysis due to the failure of complete clearance of plasma EBV DNA followed by subsequent elevation secondary to distant metastases as described further below. Therefore, the half-life clearance of plasma EBV DNA of this patient could not be determined. The overall stage distribution of these patients was stage II in 9 (20.4%), stage III in 21 (47.7%), and stage IVA in 14 (31.8%). Eighteen patients (40.1%) received induction chemotherapy before concurrent chemoradiation, as their primary tumors were close to critical organs at risks including the brainstem and/or optic nerves/chiasm. The median follow-up duration of these patients was 30.3 months (range, 6.0–74.2 months). Two (4.5%), one (2.3%), and six (13.6%) experienced local recurrence, regional recurrence, and distant metastases, respectively. One patient (2.3%) presented with stage IVA T4N0M0 disease and clinically evident dysphagia due to palsies of the last four cranial nerves by tumor compression on the brainstem developed febrile neutropenia and succumbed to subsequent aspiration pneumonia 7 weeks after completion of induction chemotherapy and concurrent chemoradiation, despite feeding tube insertion, potent broad-spectrum antibiotics, and aggressive ventilation support. His plasma EBV DNA was normalized after completion of chemotherapy and start of concurrent chemoradiation. Another three patients (6.8%) died of progressive disease of their NPC. The 3-years DMFS, PFS, and OS were 76.9, 70.1, and 88.3%, respectively, and the median DMFS, PFS, OS were 29.8, 29.4, and 30.3 months, respectively.

**Table 1:** Patient baseline characteristics in the whole study population.

**Characteristic**	**No. of Patients (%)**
	**Total (*n* = 45)**
Median follow-up (months) (range)	30.3 (6.0–74.2)
Median age in years (range)	58 (20–78)
Male/female	33 (73.3)/ 12 (26.7)
**T-classification**	
T1	12 (26.7)
T2	6 (13.3)
T3	20 (44.4)
T4	7 (15.6)
**N-classification**	
N0	2 (4.4)
N1	16 (35.6)
N2	18 (40.0)
N3	9 (20.0)
**Overall Stage**	
I	0 (0)
II	9 (20.0)
III	21 (46.7)
IVA	15 (33.3)
Median pretreatment plasma EBV DNA in copies/milliliter (range)	463 (16–54,437)
Stage II	282 (16–987)
Stage III	339 (20–54,437)
Stage IVA	1,275 (43–16,125)
Mean/ median half-life of EBV clearance (days) (range)	9.82/7.92 (0.9–40.77^*^)
Median gross tumor volume of the primary tumor (GTV_P) (cm^3^) (range)	19.0 (3.6–171.5)
Median gross tumor volume of the positive neck nodes (GTV_N) (cm^3^) (range)	9.7 (0.7–62.2)
Median gross tumor volume of the primary tumor and the positive neck nodes (GTV_P+N) (cm^3^) (range)	35.6 (5.6–173)
Concurrent chemoradiation only	10 (22.7)
Induction chemotherapy then concurrent chemoradiation	19 (42.2)
Concurrent chemoradiation then adjuvant chemotherapy	16 (36.4)

* *One patient did not achieve complete clearance of his plasma EBV DNA despite induction chemotherapy and radical chemoradiation with subsequent development of distant metastases. Therefore, the half-life clearance of his plasma EBV DNA could not be determined*.

*EBV DNA, Epstein–Barr virus deoxyribonucleic acid; ECOG, Eastern Cooperative Oncology Group*.

The median and the mean half-life clearance of plasma EBV DNA in the whole study population was 7.92 and 9.82 days, respectively. The ROC AUC for half-life of plasma EBV DNA clearance for predicting disease progression was 0.62 [95% CI: (0.38; 0.87)]. Patients were stratified into two groups based on their half-life clearance of plasma EBV DNA. After ROC analysis, the cutoff value of 15 days as the half-life clearance of plasma EBV DNA was determined as a stratifying factor for subsequent survival analyses with the corresponding sensitivity and specificity of 50.0 and 88.9%, respectively. With this as the cutoff, 36 (81.8%) and 8 (18.2%) patients had their half-life plasma EBV DNA clearance of ≤15 and >15 days, respectively. The dispositions of these 44 patients dichotomized by the half-life clearance of plasma EBV DNA are shown in Table 2.

**Table 2:** Patient baseline characteristics stratified by half-life clearance of plasma EBV DNA.

**Characteristic**	**No. of Patients (%)**			***p***
	**Total (*n* = 44)**	**Half-life of EBV clearance**		
		**≤15 days** **(*n* = 36)**	**>15 days** **(*n* = 8)**	
Median follow-up (months) (range)	30.3 (6.0–74.2)	30.8 (16.3–74.2)	28.9 (6.0–51.5)	0.39
Median age in years (range)	58 (20–78)	57.5 (20–74)	63 (46–78)	0.27
Male/female	32 (72.7)/ 12 (27.3)	26 (72.2)/10 (27.8)	6 (75)/2 (25)	0.93
**T-classification**				0.76
T1	11 (25.0)	10 (27.8)	1 (12.5)	
T2	6 (13.6)	5 (13.9)	1(12.5)	
T3	20 (45.5)	16 (44.4)	4(50.0)	
T4	7 (15.9)	5 (13.9)	2 (5.0)	
**N-classification**				0.24
N0	2 (4.5)	1 (2.8)	1 (12.5)	
N1	16 (36.4)	15 (41.7)	1 (12.5)	
N2	18 (40.9)	13 (36.1)	5 (62.5)	
N3	8 (18.2)	7 (19.4)	1 (12.5)	
**Overall Stage**				0.28
II	9 (20.5)	9 (25.0)	0 (0)	
III	21 (47.7)	16 (44.4)	5 (62.5)	
IVA	14 (31.8)	11 (30.6)	3 (37.5)	
Median pretreatment plasma EBV DNA in copies/milliliter (range)	436.5 (16–54,437)	396.5 (16–34,000)	1,033 (43–54,437)	0.39
Stage II	282 (16–987)	282 (16–987)	Inapplicable	
Stage III	339 (20–54,437)	317.5 (20–34,000)	683 (105–54,437)	0.24
Stage IVA	1,107.5 (43–11,563)	940 (79–11,563)	1,383 (43–1,538)	0.69
Mean/ median half-life of EBV clearance (days) (range)	9.82/7.92 (0.9–40.77)	6.64/6.45 (0.9–13.3)	24.09/20.73 (16.9–40.77)	<0.001
Median gross tumor volume of the primary tumor (GTV_P) (cm^3^) (range)	19.7 (3.6–171.5)	18.5 (3.6–107.3)	31.8 (9.8–171.5)	0.22
Median gross tumor volume of the positive neck nodes (GTV_N) (cm^3^) (range)	9.5 (0.7–62.2)	9.5 (0.7–62.2)	11.4 (1.5–55.3)	0.73
Median gross tumor volume of the primary tumor and the positive neck nodes (GTV_P+N) (cm^3^) (range)	35.6 (5.6–173)	33.45 (5.6–111.3)	37.9 (30.5–173)	0.09
Concurrent chemoradiation only	10 (22.7)	10 (27.8)	0 (0)	0.09
Induction chemotherapy then concurrent chemoradiation	18 (40.9)	14 (38.9)	4 (50.0)	0.56
Concurrent chemoradiation then adjuvant chemotherapy	16 (36.4)	12 (33.3)	4 (50.0)	0.69

*EBV DNA, Epstein–Barr virus deoxyribonucleic acid; ECOG, Eastern Cooperative Oncology Group*.

In our study, 11 (25.0%) patients had initial rise in plasma EBV DNA followed by subsequent continuous decline (Supplementary Figure 1). Three (37.5%) patients with half-clearance of >15 days had an initial rise in plasma EBV DNA, compared to 8 (22.2%) patients with half-life clearance of ≤15 days (*p* = 0.367).

### Treatment Outcomes of Patients With Half-Life Clearance of Plasma EBV DNA >15 vs. ≤15 Days

**Figure 2:**
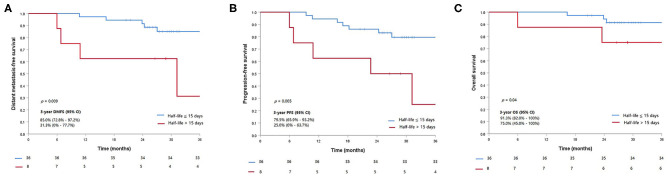
Kaplan–Meier curves of the prespecified survival endpoints including **(A)** distant metastasis-free survival, **(B)** progression-free survival, and **(C)** overall survival, stratified by the half-life clearance of plasma Epstein–Barr virus (EBV) DNA (>15 vs. ≤15 days).

Five out of eight patients (62.5%) with half-life clearance of their plasma EBV DNA >15 days subsequently developed progressive disease as compared to 7 of 36 patients (19.4%) (*p* = 0.013). Similarly, 50% of patients (four patients) with half-life clearance of >15 days as compared to 13.9% (five patients) with half-life clearance of ≤15 days suffered from distant metastasis later (*p* = 0.02). Patients with half-life clearance of their plasma EBV DNA >15 days had a shorter 3-years DMFS (*p* = 0.009), PFS (*p* = 0.005), and OS (*p* = 0.02) as compared to their counterparts with half-life clearance of ≤15 days (Table 3). The Kaplan–Meier estimates of the prespecified survival endpoints were also shown, respectively (Figure 2). Intriguingly, no statistical significance could be observed in half-life of plasma EBV DNA clearance between patients who received induction chemotherapy and their counterpart (Supplementary Table 1 and Figures 2A,B).

**Table 3:** Impact of half-life clearance of plasma EBV DNA on various prespecified survival endpoints.

	**Half-life clearance of plasma EBV DNA**		
	**Half-life ≤15 days**	**Half-life >15 days**	***P***
**Distant Metastasis-Free Survival**			0.009
3-years rate	85.0%	31.3%	
95% confidence interval	72.8–97.2%	0–77.7%	
Median (months)	NR	31.20	
95% confidence interval	NA	0.93–61.47	
Mean (months)	66.17	24.41	
95% confidence interval	59.67–72.68	15.09–33.94	
**Progression-Free Survival**			0.005
3-years rate	79.5%	25.0%	
95% confidence interval	65.9–93.2%	0–63.7%	
Median (months)	NR	22.70	
95% confidence interval	NA	3.88–41.52	
Mean (months)	62.66	23.04	
95% confidence interval	55.00–70.32	14.15–31.93	
**Overall Survival**			0.024
3-years rate	91.3%	75.0%	
95% confidence interval	82.0–100%	45.0–100%	
Median (months)	NR	38.63	
95% confidence interval	NA	16.85–60.42	
Mean (months)	69.61	37.48	
95% confidence interval	64.68–74.55	25.80–49.15	

*NA, not available; NR, not reached*.

The prognostic significance of half-life of 15 days and survival endpoints were further evaluated in Cox proportional hazard models with univariable and multivariable analyses for DMFS, PFS, and OS (Table 4). Univariable and multivariable analyses showed that half-life clearance of plasma EBV DNA was the only prognostic factor of DMFS [HR, 4.91; 95% CI: (1.31; 18.39), *p* = 0.01]. On the other hand, both univariable and multivariable analyses identified that half-life clearance of plasma EBV DNA >15 days (*p* = 0.01 and *p* = 0.02, respectively) and GTV_P+N (*p* = 0.01 and *p* = 0.02, respectively) were all significantly prognostic of PFS. Only half-life clearance of plasma EBV DNA was prognostic of a worse OS in both univariable and multivariable analyses (HR, 5.24, 95% CI: (1.06; 26.05), *p* = 0.04].

**Table 4:** Univariable and multivariable analyses of variables prognostic of distant metastasis-free survival, progression-free survival, and overall survival.

	**Univariable analysis**		**Multivariable analysis**	
	**Hazard ratio**	***p***	**Hazard ratio**	***P***
	**(95% confidence interval)**		**(95% confidence interval)**	
**Distant Metastasis-Free Survival**				
Age (every 1-year increment)	1.01 (0.94–1.09)	0.75	–	–
Male (vs. female)	0.77 (0.19–3.08)	0.71	–	–
*T* stage (3–4) vs. (1–2)	1.47 (0.37–5.88)	0.59	–	–
*N* stage (2–3) vs. (0–1)	1.46 (0.36–5.84)	0.59	–	–
Overall stage			–	–
II (as reference)	1.00	–	–	–
III	1.20 (0.23–6.19)	0.83	–	–
IVA	0.72 (0.10–5.10)	0.74	–	–
GTV_P	1.01 (1.00–1.03)	0.15	–	–
GTV_N	1.01 (0.97–1.05)	0.61	–	–
GTV_P+N	1.01 (1.00–1.03)	0.08	1.00 (0.99–1.02)	0.35
Half-life of EBV clearance (>15 days)	4.91 (1.31–18.39)	0.01	4.91 (1.31–18.39)	0.01
**Progression-Free Survival**				
Age (every 1-year increment)	1.04 (0.98–1.10)	0.22		
Male (vs. female)	0.52 (0.16–1.64)	0.26	–	–
*T* stage (3–4) vs. (1–2)	1.50 (0.45–4.98)	0.51	–	–
*N* stage (2–3) vs. (0–1)	1.46 (0.44–4.86)	0.54	–	–
Overall stage			–	–
II	Reference	–	–	–
III	1.83 (0.38–8.86)	0.45	–	–
IVA	1.10 (0.18–6.57)	0.92	–	–
GTV_P	1.01 (1.00–1.03)	0.02	0.98 (0.95–1.01)	0.15
GTV_N	1.02 (0.99–1.56)	0.12	–	–
GTV_P+N	1.02 (1.01–1.03)	0.01	1.01 (1.00–1.03)	0.02
Half-life of EBV clearance (>15 days)	4.46 (1.41–14.11)	0.01	5.14 (1.28–22.73)	0.02
**Overall Survival**				
Age (every 1-year increment)	1.08 (0.98–1.18)	0.11	–	–
Male (vs. female)	0.72 (0.13–3.93)	0.70	–	–
*T* stage (3–4) vs. (1–2)	1.42 (0.26–7.76)	0.69	–	–
*N* stage (2–3) vs. (0–1)	1.38 (0.25–7.56)	0.71	–	–
Overall stage			–	–
II	Reference	–	–	–
III	1.32 (0.14–13.00)	0.81	–	–
IVA	1.52 (0.14–16.82)	0.73	–	–
GTV_P	1.02 (1.00–1.03)	0.07	0.99 (0.95–1.03)	0.54
GTV_N	1.02 (0.97–1.06)	0.51	–	–
GTV_P+N	1.02 (1.00–1.04)	0.03	1.01 (1.00–1.03)	0.16
Half-life of EBV clearance (>15 days)	5.24 (1.06–26.05)	0.04	5.24 (1.06–26.05)	0.04

^*^Only covariates with a p < 0.1 in the univariable analysis were considered in the multivariable analysis (backward elimination).

The excluded patient was a 53-years-old patient diagnosed of stage IVA (T1N3M0) non-metastatic NPC with a baseline pretreatment plasma EBV DNA of 16,125 copies/ml. He received three cycles of induction chemotherapy (gemcitabine plus cisplatin) followed by radical concurrent chemoradiation. His plasma EBV DNA was initially decreasing although fluctuations were seen. However, complete clearance of his plasma EBV DNA could not be achieved despite active treatment without any interruption of his induction chemotherapy and concurrent chemoradiation (Supplementary Figure 3), and thus, the half-life of his plasma EBV DNA clearance could not be determined for further statistical analysis. PET-CT scan at 8 weeks after completion of radical chemoradiation demonstrated multiple bilateral tiny lung nodules and prominent mediastinal nodes of undetermined nature. Another PET-CT scan performed 11 weeks later showed further enlargement of mediastinal nodal metastasis and lung metastasis accompanied by further elevation of plasma EBV DNA, which confirmed the development of distant metastases (Supplementary Figures 4A–D).

## Discussion

There have been numerous studies investigating the roles of plasma EBV DNA in NPC. It has been so far the most accurate biomarker for NPC detection, monitoring, and prognostication (5–11). Elimination kinetics of plasma EBV DNA during radiation therapy was also studied previously (12). Lo et al. demonstrated the median half-life of plasma EBV DNA clearance was 3.8 days for irradiated patients in the period between the third and 7th week of radiotherapy and reported the initial rise in plasma EBV DNA following its liberation from therapy-induced tumor cell death (12). The prognostic effect of its clearance rate on survival was also confirmed later (13–15). Patients with more rapid reduction in plasma EBV DNA had better tumor response and survival outcomes. However, these few studies were limited to recurrent or metastatic diseases only.

Recent emphasis has also focused on posttreatment EBV DNA as prognosticators (6, 10, 11, 15, 19, 20, 26–28). Chen et al., in their recent study, further demonstrated the role of plasma EBV DNA in the detection of early disease recurrence among patients with NPC after treatment (29). Leung et al. showed that detectable plasma EBV DNA titers at completion of 4 weeks of concurrent chemoradiation or radiation (mid-EBV DNA) was prognostic of a worse PFS and OS (9). Another study by Lertbutsayanukul et al. also echoed the finding that undetectable mid-EBV DNA was prognostic of PFS and OS (21). These two studies indicated that prognosticative information from plasma EBV DNA could be obtained earlier at the midcourse of therapy (16). This still begs to the question whether further upfront and earlier plasma EBV DNA measurement right after treatment commencement and before the midcourse of therapy is still prognostic or not. To the best of our knowledge, there has been no publication on the role of EBV DNA clearance on survival prognostication in the current contemporary era when the 8th edition of AJCC/UICC staging classification and IMRT are fully implemented. Our findings echoed with the results of a retrospective study in China, which reported the plasma EBV DNA clearance in response to treatment, with measurement of plasma EBV DNA per 3-weeks cycle of induction chemotherapy (30). On the contrary, our study is the first prospective observational study investigating the prognosticative role of plasma EBV DNA clearance rate on various predefined survival endpoints on the basis of full IMRT implementation and the 8th edition of AJCC staging system. We measured plasma EBV DNA at weekly intervals until it was undetectable. Half-life clearance of plasma EBV DNA was obtained, which better reflected the clearance kinetics in spite of our relatively small sample size.

Besides, we measured plasma EBV DNA consistently by using the same assay in the same institution for all patients. The method of EBV DNA assay was in line with those used by Le et al. and a recent NPC screening programme in Hong Kong (31, 32). Plasma EBV DNA was measured by the same assay in a single institution on the same day of blood sampling to avoid any inconsistency and error due to delayed processing. All samples were repeated twice on the same day by the same assay for accurate quantification, and the results showed that the discrepancy was <2% for all repeated samples (24).

We demonstrated that half-life clearance of plasma EBV DNA >15 days was prognostic of DMFS and PFS. Setting the half-life as 15 days was based on our prior ROC analysis. Our results implied that 15 days (i.e., about 2 weeks earlier than the midcourse of concurrent chemoradiation or radiation alone) could allow us to identify early poor responders and performers, which may need more prompt and duly investigations to rule out recurrence/metastasis and earlier interventions to reduce the chance of later relapse. We also identified that 50% (i.e., four out of eight) patients whose half-life clearance of plasma EBV DNA >15 days suffered from progressive disease. Of them, one received induction chemotherapy for his stage IVA disease. It alerts us to exercise earlier scrutiny to detect out-of-radiation-field treatment failure with distant metastasis, which can be clinically occult as these patients' primary tumors and regional nodes can be responding well to concurrent chemoradiation. Active vigilance should also be taken for those who receive induction chemotherapy since it is not guaranteed that all patients respond to induction chemotherapy. After all, the best induction chemotherapy regimen is still yet to be determined (33, 34).

Our results also provided information to clinicians to consider additional or more intensified treatment for high-risk patients with half-life clearance >15 days. However, several issues need to be addressed in future studies. First of all, the benefits of adaptive radiotherapy or more intensified chemotherapy for those with persistently detectable plasma EBV DNA are still controversial (35–39). While the Taiwan study demonstrated an OS benefit with additional 1-year therapy with tegafur-uracil in those who had detectable EBV DNA titers taken at 1 week after completion of radiation therapy, the Hong Kong NPC-0502 study investigating six cycles of adjuvant chemotherapy with gemcitabine and cisplatin in those patients with detectable EBV DNA titers at 6 weeks after completion of concurrent chemoradiation failed to prolong OS (37, 38). The combined phase II and III NRG-HN001 trial (ClinicalTrials.gov NCT02135042) to test the feasibility of using plasma EBV DNA following IMRT to personalize the treatment regimen is heavily awaited (39). One of the study objectives is to evaluate whether replacing adjuvant cisplatin and 5-FU with gemcitabine and paclitaxel for patients with detectable plasma EBV DNA following IMRT would bring superior PFS. That said, the equipoise between survival prolongation and treatment-related toxicities brought by additional chemotherapy is another unresolved issue. In addition, whether patients with more rapid half-life clearance of <15 days during treatment could be spared from de-escalated and more customized therapy in their remaining course of radical treatment remains unknown. It is highly expected that ongoing clinical trials could provide a clearer answer. The other limitation of our study is the relatively short follow-up duration. It was noted that the median survival end points were not reached in the subgroups with half-life >15 days, due to a relative lack of failure events. Nevertheless, it could also be attributed to our excellent treatment outcomes (24). A longer follow-up period will certainly provide us a mature result and pose the basis for future studies investigating the best treatment approaches for these high-risk patients.

## Conclusion

Our study results, in the era of IMRT with the launch of the latest staging system, were distinct from other previous studies using plasma EBV DNA kinetics as prognosticators. The half-life clearance rate of plasma EBV DNA during the very early phase of radical treatment was prognostic in patients with previously untreated non-metastatic NPC. Future studies are warranted to investigate if early change in treatment strategy secondary to an unsatisfactory decline of plasma EBV DNA would impact on survival.

## Data Availability Statement

The datasets presented in this article are not readily available due to national legislation and the terms of the study's ethics approval do not allow dataset sharing outside of the institutions participating in the analysis. Requests to access the datasets should be directed to Victor Ho-Fun Lee, vhflee@hku.hk.

## Ethics Statement

The studies involving human participants were reviewed and approved by Institutional Review Board of the University of Hong Kong/Hospital Authority Hong Kong West Cluster. The patients/participants provided their written informed consent to participate in this study.

## Author's Note

Part of the results of this manuscript was presented as an abstract in ASTRO Annual Meeting 2019 on September 15 to 18, 2019.

## Author Contributions

S-KC, S-YC, HC, DK, and VL designed the study. S-KC, HC, and VL performed the statistical analysis. All authors contributed patient data, participated in reviewing, improving statistical analysis and manuscript, read, and approved the final manuscript. All authors contributed to the article and approved the submitted version.

## Conflict of Interest

The authors declare that the research was conducted in the absence of any commercial or financial relationships that could be construed as a potential conflict of interest.
