# Supplementary material for: Prognostication of Half-Life Clearance of Plasma EBV DNA in Previously Untreated Non-metastatic Nasopharyngeal Carcinoma Treated With Radical Intensity-Modulated Radiation Therapy
Source: Front Oncol. 2020 Aug 21;10:1417. doi: 10.3389/fonc.2020.01417 (PMC7472777; doi:10.3389/fonc.2020.01417)
Supplement: Supplementary file 1 [file Data_Sheet_1.docx]

**Supplementary Material**

**Prognostication of Half-Life Clearance of Plasma EBV DNA in Previously Untreated Non-Metastatic Nasopharyngeal Carcinoma Treated with Radical Intensity-Modulated Radiation Therapy**

**(I) Determination of plasma EBV DNA titers**

In brief, four milliliters (ml) of peripheral blood was drawn and placed in an EDTA tube. All samples were immediately stored in a 4 degree Celsius refrigerator after blood taking from all patients and they were processed for subsequent EBV DNA extraction within 4 hours of blood taking from patients in the single laboratory of our institution. A total of about 400 to 800 microliters of plasma samples were used for DNA extraction by a QIAamp Blood Kit (Qiagen, Hilden, Germany). The exact amount of plasma was determined for calculation of EBV DNA genome copies. Circulating EBV DNA concentrations were measured using a real-time quantitative polymerase chain reaction (PCR) system with ABI Prism® 7000 Sequence Detection System (Applied Biosystems, USA) that amplified a DNA segment in the *Bam*HI-W fragment region of the EBV genome. All plasma DNA samples were also subject to real-time PCR analysis for the β*-globin* gene, which gave a positive signal on all tested samples. Multiple controls without templates were also included in each analysis as negative controls. All samples were repeated twice on the same day by the same assay for accurate quantification and the results showed that the discrepancy was less than 2% for all repeated samples. All results were expressed as EBV DNA genome copies per ml with accuracy to the nearest 0.1 copies/ml. Undetectable plasma EBV DNA meant 0 copies/ml and they were used interchangeably in the main text and the Supplemental Material.

**(II) Imaging examinations for NPC staging**

One independent oncologist and another independent radiologist who were specialized in head and neck radiology and blinded to study treatment details reviewed all PET-CT and MRI images to determine the stage and tumor extent based on the 7^th^ edition of American Joint Committee (AJCC)/Union for International Cancer Control (UICC) Staging Classification (TNM) for treatment decision. Re-staging based on the 8^th^ edition of AJCC/UICC Staging Classification was blindly performed again for subsequent analyses in this study. Any stage discrepancies were resolved by consensus. All of the imaging scans were performed by the same PET-CT and MRI scanners at Department of Diagnostic Radiology, Queen Mary Hospital, The University of Hong Kong throughout the whole study period.

**(III) PET-CT scan protocol**

Whole body [18F]fluorodeoxyglucose (18F-FDG) PET-CT was performed with a combined PET-CT scanner (Discovery VCT, 64 multislice spiral CT; GE Healthcare Bio-Sciences Corp) using a standardized protocol. All patients were immobilized in a supine treatment position by a custom-made thermoplastic head and neck cast before scanning. The scanning range was from the vertex of brain to the upper thigh. After six hours of fasting, 220 to 370 MBq [4.8 MBq/kilogram (kg)] of body weight-adjusted 18F-FDG was administered intravenously. After a 60-minute uptake time, whole-body emission PET scans were obtained with seven bed positions. Attenuation-corrected PET images with CT data were reconstructed with an ordered-subset expectation maximization iterative reconstruction algorithm (14 subsets and two iterations) and fused with CT images (Advanced Workstation 4·3; GE Healthcare Bio-Sciences). The CT imaging parameters were as follows: 120kVp; 200 to 400 mA; 0.5 second per CT rotation; pitch 0.984:1; and 2.5 mm intervals, with or without 60 to 100 ml (1.5 ml/kg of body weight) intravenous contrast medium.

**(IV) MRI techniques**

MRI scanning was performed on the following day after PET-CT scanning with a 3.0-T MR scanner (Achieva 3·0T, Philips Healthcare, Best, The Netherlands), utilizing a 16-channel neurovascular coil. Image acquisition was made in axial plane from suprasellar region cranially to lung apices caudally. Four standard sequences were performed: a) axial T1-weighted turbo spin echo (TSE) [repetition time/echo time (TR/TE) = 454/9·2 milliseconds (ms); turbo factor = 3; FOV = 230 × 230 millimeter (mm); matrix = 672 × 672; slice number = 32; slice thickness = 3 mm; intersection gap = 0.3 mm]; b) axial T2-weighted short TI inversion recovery (STIR) [TR/TE = 4644/60 ms; field-of-view [FOV] = 230 × 230 mm; matrix = 672 × 672; slice number = 32; slice thickness = 3 mm; intersection gap = 0·3 mm]; c) coronal T2-weighted STIR [TR/TE = 4644/60 ms; FOV = 230 × 230 mm; matrix = 480 × 480; slice number = 32; slice thickness = 3 mm; intersection gap = 0.3 mm]; d) 3D T1-weighted turbo-field- echo (TFE) post-contrast scan [TR/TE = 4.8/2.4 ms; flip angle = 100; FOV = 230 × 230 mm; matrix = 640 × 640; slice number = 319; slice thickness = 0.72 mm]. Intravenous bolus injection of 0.1 mmol/kg of body weight gadopentetate dimeglumine was then administered at 1.5 ml per second for post-contrast acquisition.

**(V) Intensity-modulated radiation therapy (IMRT) planning protocol**

Every patient was immobilized in the supine position during 18F-FDG PET-CT acquisition and actual treatment by using a thermoplastic head and neck cast. A customized mouthguard was fabricated for better immobilization. MRI images mentioned above were co-registered with the planning PET-CT images for dedicated delineation of the target volumes and organs-at-risk (OARs). OARs, including brainstem, spinal cord, globes, optic nerves, optic chiasm, lenses, temporomandibular joints, temporal lobes, auditory nerves, cochleae, mandible, oral cavity, larynx, parotid glands and vestibules were first contoured. Then gross tumor volumes (GTV) of both the primary tumor and the radiologically involved cervical nodes were outlined. Subsequently, the clinical target volume (CTV-70) for the microscopic disease spread and planning target volume containing CTV-70 with a 5-mm margin (PTV-70) to take into account physiological body motion and set-up errors were generated respectively. Another CTV-66 encompassing the high-risk areas including the posterior half of the maxillary sinuses, nasal cavities, parapharyngeal spaces, styloid processes, basiocciput, basisphenoid, clivus, foramina rotunda and ovale, pterygopalatine fossae, pterygomaxillary fissures, infraorbital fissures, cavernous sinuses, and level Ib and V nodal stations were also outlined subsequently. A corresponding PTV-66 with a 3-mm margin encompassing the CTV-66 was created by Boolean operations of the treatment planning system (Eclipse version 8.0 to 10.0 software, Eclipse Treatment Planning System, Palo Alto, CA, USA), which was also used for IMRT planning using Analytical Anisotropic Algorithm. All the targets and OARs delineation were approved by senior radiation oncologists (Victor Lee, Dora Kwong and To-Wai Leung) before dose optimization for IMRT. During optimization, the maximum dose to the brainstem, optic nerves, and chiasm was limited to 54 Gy and less than 45 Gy to the spinal cord. Allowance was given for some locally advanced tumors in which the maximum dose to the brainstem, optic nerves and chiasm could be up to 60 Gy. Efforts were also made to limit the mean dose to the parotid glands to 26 Gy and the dose to the lenses and temporal lobes to as low as could reasonably be achieved without compromising dose coverage to the PTVs. A dose of 70 Gy was prescribed to the PTV-70 and 66 Gy to the PTV-66 in 33 fractions delivered by the simultaneous accelerated radiation therapy technique (SMART). If there was no clinical nodal disease in the patients’ lower neck, either an extended IMRT field or a separate anterior field matched with a monoisocentric technique to the IMRT field above was employed based on the oncologist’s preference and 66Gy in 33 fractions were prescribed for the anterior neck field if present. All IMRT planning, dose optimization and quality assurance was performed by a certified medical physicist (Sherry Ng) and all IMRT plans fulfilled acceptance criteria with at least 95% of PTVs having received the prescribed dose, the maximum dose of PTVs limited to 107% or below and the maximum dose of organs-at-risk within tolerance limits according to International Commission on Radiation Units and Measurements (ICRU) criteria. They were then approved by senior radiation oncologists (Victor Lee, Dora Kwong and To-Wai Leung) before IMRT commencement. Positional verification with on-board imaging was performed before and then daily before the first 3 fractions of IMRT followed by weekly afterwards during the whole course of IMRT, to track any anteroposterior and lateral body displacements.

**(VI) Chemotherapy regimens and schedules**

Treatment was based on 7^th^ and 8^th^ edition of AJCC/UICC system and Eastern Cooperative Oncology Group (ECOG) performance status and medical comorbidities of each patient. In general, patients with stage I and II disease received IMRT alone while stage III to IVB received concurrent chemoradiation with either adjuvant or induction chemotherapy. Concurrent chemotherapy using intravenous infusional cisplatin (100mg/m^2^) was given on the first day of IMRT every 3 weeks for up to three cycles. Adjuvant chemotherapy with cisplatin (80mg/m^2^) on day 1 and 5-FU (1000mg/m^2^) from day 1 to 4 every four weeks for three cycles was started at four weeks following completion of IMRT. Patients who had their primary tumors close to critical OARs would receive three cycles of induction chemotherapy (cisplatin 100mg/m^2^ on day 1 and either 5-FU 1000mg/m^2^ from day 1 to 5 or gemcitabine 1000mg/m^2^ on day 1 and day 8, given every 3 weeks) before concurrent chemoradiation, in an attempt to achieve satisfactory tumor shrinkage so that a radical radiation dose could be delivered to the tumors with more sparing of these OARs from radiation. Those with bulky cervical nodal (≥ 3cm in diameter) stage II disease were also given concurrent chemoradiation only, at the discretion of the treating oncologist.

**(VII) Post-IMRT surveillance and follow-up**

Eight weeks following completion of IMRT, all patients underwent nasoendoscopy again with routine 6-site random nasopharyngeal biopsies at both roofs, lateral and posterior walls of the nasopharynx. If residual tumors were noted histologically, they needed another endoscopy and biopsies two weeks later as positive histological findings may undergo spontaneous remission with time, as we reported previously. Another endoscopy and biopsies would be performed again at 12^th^ week after completion of IMRT if there was still residual tumor at 10^th^ week after completion of IMRT. Local persistence, as we defined for more than 15 years in our institution, was tumor persistence in nasopharyngeal biopsies at 12 weeks after IMRT and patients would receive salvage treatment for instance intracavitary brachytherapy boost, stereotactic radiotherapy etc. If all 6-site biopsies were negative, patients were considered to have complete local remission. They also had plasma EBV DNA checked again on the same day of nasoendoscopy at eight weeks after IMRT completion. If their EBV DNA was still > 0 copies/ml, it would be repeated every four weeks thereafter until it was undetectable or until it was proven to have persistent local or regional disease, or distant metastasis. For patients with complete local remission, they would have regular follow-up every two to three months for any relapse and complications, as well as MRI scan every three to four months and PET-CT scan if clinically suspicious of relapse.

**(VIII) Survival end points**

Prespecified survival end points in this study include distant metastasis-free survival (DMFS), progression-free survival (PFS), overall survival (OS). DMFS was defined as the time from date of diagnosis of NPC to the date of distant metastasis or death from any cause. PFS was defined as the time from date of diagnosis of NPC to the date of any form of disease progression or death from any cause. OS was defined as the time from the date of diagnosis of NPC to the date of death from any cause.

**Supplementary Table 1.** Patient characteristics stratified by the use of induction chemotherapy before concurrent chemoradiation

| **Characteristic** | **No. of Patients (%)** | | | ***p*** |
| --- | --- | --- | --- | --- |
|  | **Total (*n* = 44)** | **Induction chemotherapy**  **(*n* = 18)** | **No induction chemotherapy**  **(*n* = 26)** |  |
| Median follow-up (months) (range) | 30.3  (6.0–74.2) | 28.1 (6.0–59.2) | 38.0 (16.3–74.2) | 0.10 |
| Median age in years (range) | 58 (20–78) | 57.5 (20–78) | 58.5 (42–74) | 0.84 |
| Male/female | 32 (72.7) /  12 (27.3) | 13(72.2)/5(27.8) | 19(73.1)/7(26.9) | 0.06 |
| T-classification |  |  |  | 0.01 |
| T1 | 11 (25.0) | 2 (11.1) | 9 (34.6) |  |
| T2 | 6 (13.6) | 2 (11.1) | 4(15.4) |  |
| T3 | 20 (45.5) | 7 (38.9) | 13(50.0) |  |
| T4 | 7 (15.9) | 7 (38.9) | 0 (0) |  |
| N-classification |  |  |  | <0.001 |
| N0 | 2 (4.5) | 2 (11.1) | 0 (0) |  |
| N1 | 16 (36.4) | 1 (5.6) | 15 (57.7) |  |
| N2 | 18 (40.9) | 8 (44.4) | 10 (38.5) |  |
| N3 | 8 (18.2) | 7 (38.9) | 1 (3.8) |  |
| Overall stage |  |  |  | <0.001 |
| I | 0 (0) | 0 (0) | 0 (0) |  |
| II | 9 (20.5) | 0 (0) | 9 (34.6) |  |
| III | 21 (47.7) | 5 (27.8) | 16 (61.5) |  |
| IVA | 14 (31.8) | 13 (72.2) | 1 (3.8) |  |
| Median pretreatment plasma EBV DNA in copies/milliliter (range) | 436.5  (16–54437) | 592  (43–11563) | 396.5  (16–54437) | 0.06 |
| Stage II | 282 (16–987) | Inapplicable | 282 (16-987) | - |
| Stage III | 339  (20–54437) | 309  (90–721) | 546.5  (20–54437) | 0.40 |
| Stage IVA | 1107.5  (43–11563) | 1275  (43–11563) | 868  (868) | 0.77 |
| Mean/ median half-life of EBV clearance (days) (range) | 9.82/7.92  (0.9–40.77) | 10.24/8.16 (0.9–40.77) | 10.35/7.97 (1.35–33.0) | 0.71 |
| Median gross tumor volume of the primary tumor (GTV_P) (cm^3^) (range) | 19.7  (3.6–171.5) | 33.1 (3.6–171.5) | 14.65  (4.5–42) | 0.02 |
| Median gross tumor volume of the positive neck nodes (GTV_N) (cm^3^) (range) | 9.5  (0.7–62.2) | 6.7  (1.5–62.2) | 9.85  (0.7–55.3) | 0.47 |
| Median gross tumor volume of the primary tumor and the positive neck nodes (GTV_P+N) (cm^3^) (range) | 35.6  (5.6–173) | 62.6  (7.1–173) | 32.45  (5.6–72) | <0.01 |

*EBV DNA, Epstein-Barr virus deoxyribonucleic acid; ECOG, Eastern Cooperative Oncology Group.*

**
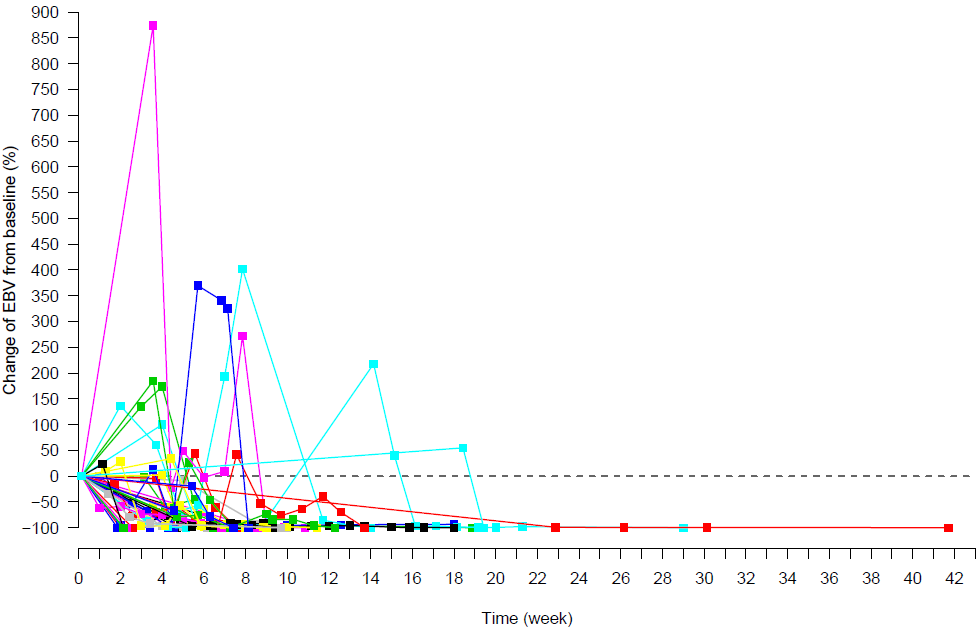
**

**Supplementary Figure 1.**

Changes of plasma EBV DNA during IMRT with or without concurrent chemoradiation and/or adjunct (induction or adjuvant) chemotherapy in the whole study population.


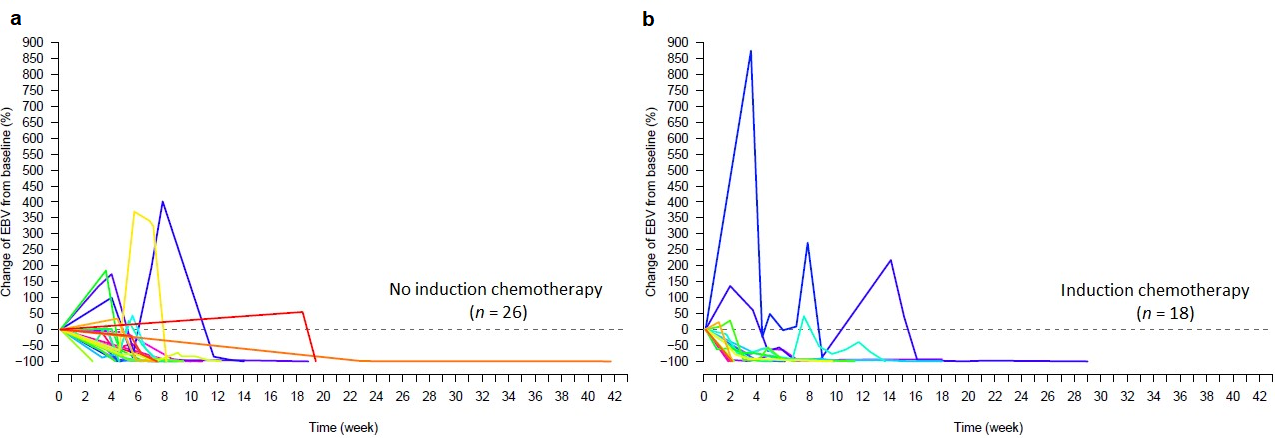


**B**

**A**

**Supplementary Figure 2.** Changes of plasma EBV DNA of patients stratified by the use of induction chemotherapy in patients who did not receive induction chemotherapy (A) and patients who received induction chemotherapy (B).


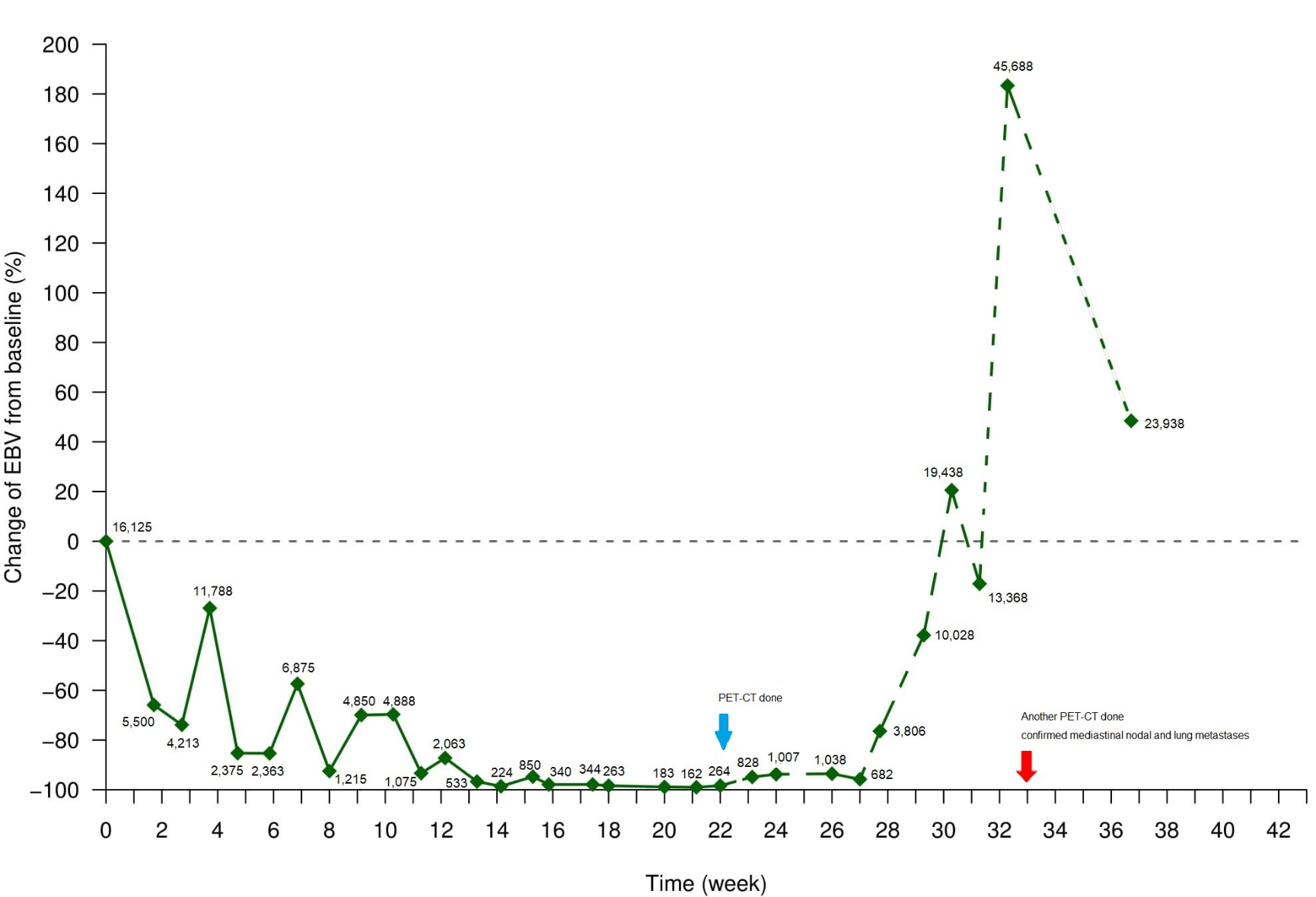


**Supplementary Figure 3.** Trend of plasma EBV DNA of the excluded patient who could not achieve complete clearance of his plasma EBV DNA despite definite intensive radical treatment with induction chemotherapy followed by concurrent chemoradiation. PET-CT scan at 8 weeks after completion of radical chemoradiation therapy (i.e. week 22 indicated by the blue arrow) showed undetermined multiple bilateral tiny lung lesions and mediastinal lymph nodes. Another PET-CT scan performed at week 33 (red arrow) showed further enlargement of the mediastinal lymph nodes and lung lesions accompanied by further elevations of his plasma EBV DNA (represented by the dashed line), which confirmed the development of distant metastases.


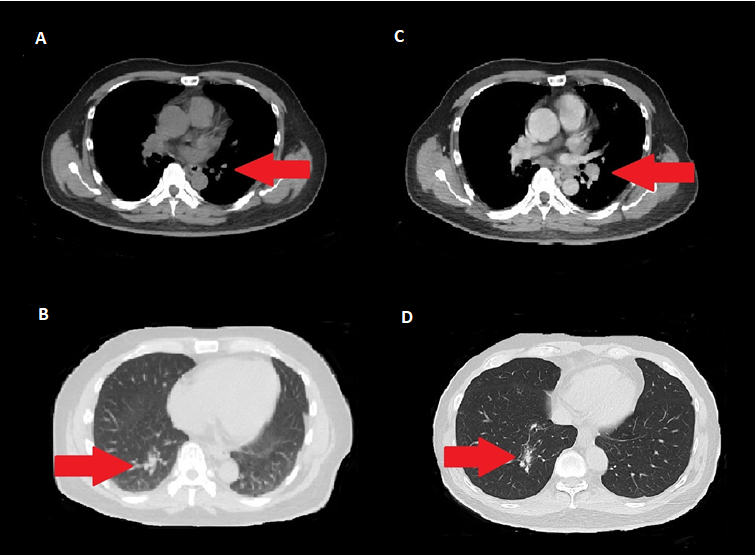


**Supplementary Figure 4.** Positron emission tomography with integrated computed tomography (PET-CT) images of the excluded patient due to failure of complete clearance of his plasma EBV DNA with subsequent development of distant metastasis. PET-CT images showing a tiny indeterminate left hilar lymph node and a suspicious nodule in his right lower lobe of lung (red arrows) (A,B) Another PET-CT scan performed 11 weeks later showing a progressively enlarged left hilar node as well as persistent right lower lung nodule (red arrows) (C,D), accompanied by a progressive rise in plasma EBV titers, confirming the development of mediastinal nodal and lung metastasis.

**REMARK Checklist**

| **Item to be reported** | | **Page no.** |
| --- | --- | --- |
| **INTRODUCTION** | |  |
| 1 | State the marker examined, the study objectives, and any pre-specified hypotheses. | 3 |
| **MATERIALS AND METHODS** | |  |
| *Patients* | |  |
| 2 | Describe the characteristics (e.g., disease stage or co-morbidities) of the study patients, including their source and inclusion and exclusion criteria. | 4 |
| 3 | Describe treatments received and how chosen (e.g., randomized or rule-based). | 4,5 |
| *Specimen characteristics* | |  |
| 4 | Describe type of biological material used (including control samples) and methods of preservation and storage. | 5 |
| *Assay methods* | |  |
| 5 | Specify the assay method used and provide (or reference) a detailed protocol, including specific reagents or kits used, quality control procedures, reproducibility assessments, quantitation methods, and scoring and reporting protocols. Specify whether and how assays were performed blinded to the study endpoint. | 5,6 |
| *Study design* | |  |
| 6 | State the method of case selection, including whether prospective or retrospective and whether stratification or matching (e.g., by stage of disease or age) was used. Specify the time period from which cases were taken, the end of the follow-up period, and the median follow-up time. | 4 |
| 7 | Precisely define all clinical endpoints examined. | 6 |
| 8 | List all candidate variables initially examined or considered for inclusion in models. | 6 |
| 9 | Give rationale for sample size; if the study was designed to detect a specified effect size, give the target power and effect size. | 4 |
| *Statistical analysis methods* | |  |
| 10 | Specify all statistical methods, including details of any variable selection procedures and other model-building issues, how model assumptions were verified, and how missing data were handled. | 6, 7 |
| 11 | Clarify how marker values were handled in the analyses; if relevant, describe methods used for cutpoint determination. | 6,7 |
| **RESULTS** | |  |
| *Data* | |  |
| 12 | Describe the flow of patients through the study, including the number of patients included in each stage of the analysis (a diagram may be helpful) and reasons for dropout. Specifically, both overall and for each subgroup extensively examined report the numbers of patients and the number of events. | 6-8 |
| 13 | Report distributions of basic demographic characteristics (at least age and sex), standard (disease-specific) prognostic variables, and tumor marker, including numbers of missing values. | 6-8 |
| *Analysis and presentation* | |  |
| 14 | Show the relation of the marker to standard prognostic variables. | 8,9 |
| 15 | Present univariable analyses showing the relation between the marker and outcome, with the estimated effect (e.g., hazard ratio and survival probability). Preferably provide similar analyses for all other variables being analyzed. For the effect of a tumor marker on a time-to-event outcome, a Kaplan-Meier plot is recommended. | 8 |
| 16 | For key multivariable analyses, report estimated effects (e.g., hazard ratio) with confidence intervals for the marker and, at least for the final model, all other variables in the model. | 8 |
| 17 | Among reported results, provide estimated effects with confidence intervals from an analysis in which the marker and standard prognostic variables are included, regardless of their statistical significance. | 7,8 |
| 18 | If done, report results of further investigations, such as checking assumptions, sensitivity analyses, and internal validation. | N/A |
| **DISCUSSION** | |  |
| 19 | Interpret the results in the context of the pre-specified hypotheses and other relevant studies; include a discussion of limitations of the study. | 9-12 |
| 20 | Discuss implications for future research and clinical value. | 9-12 |
